# The Tritryps Comparative Repeatome: Insights on Repetitive Element Evolution in Trypanosomatid Pathogens

**DOI:** 10.1093/gbe/evz017

**Published:** 2019-02-04

**Authors:** Sebastián Pita, Florencia Díaz-Viraqué, Gregorio Iraola, Carlos Robello

**Affiliations:** 1 Laboratory of Host Pathogen Interactions, Unidad de Biología Molecular, Institut Pasteur de Montevideo, Montevideo, Uruguay; 2 Sección Genética Evolutiva, Facultad de Ciencias, Universidad de la República, Montevideo, Uruguay; 3 Microbial Genomics Laboratory, Institut Pasteur Montevideo, Montevideo, Uruguay; 4 Centro de Biología Integrativa, Universidad Mayor, Santiago de Chile, Chile; 5 Departamento de Bioquímica, Facultad de Medicina, Universidad de la República, Montevideo, Uruguay

**Keywords:** trypanosomatids, Tritryps, repetitive DNA, RepeatExplorer, RepeatExplorer, transposable elements

## Abstract

The major human pathogens *Trypanosoma cruzi*, *Trypanosoma brucei*, and *Leishmania major* are collectively known as the Tritryps. The initial comparative analysis of their genomes has uncovered that Tritryps share a great number of genes, but repetitive DNA seems to be extremely variable between them. However, the in-depth characterization of repetitive DNA in these pathogens has been in part neglected, mainly due to the well-known technical challenges of studying repetitive sequences from de novo assemblies using short reads. Here, we compared the repetitive DNA repertories between the Tritryps genomes using genome-wide, low-coverage Illumina sequencing coupled to RepeatExplorer analysis. Our work demonstrates that this extensively implemented approach for studying higher eukaryote repeatomes is also useful for protozoan parasites like trypanosomatids, as we recovered previously observed differences in the presence and amount of repetitive DNA families. Additionally, our estimations of repetitive DNA abundance were comparable to those obtained from enhanced-quality assemblies using longer reads. Importantly, our methodology allowed us to describe a previously undescribed transposable element in *Leishmania major* (TATE element), highlighting its potential to accurately recover distinctive features from poorly characterized repeatomes. Together, our results support the application of this low-cost, low-coverage sequencing approach for the extensive characterization of repetitive DNA evolutionary dynamics in trypanosomatid and other protozoan genomes.

## Main Text

Collectively known as the “Tritryps,” the unicellular mono-flagellated protozoan parasites *Trypanosoma cruzi, Trypanosoma brucei*, and *Leishmania major* are the causative agents of American trypanosomiasis, cutaneous leishmaniasis, and African tryrpanosomiasis, respectively. These dixenous parasites belong to the family *Trypanosomatidae*, within the order *Kinetoplastida* (Votýpka et al. 2015). Despite Tritryps share many general characteristics which are used as distinctive taxonomic markers (i.e., their unique mitochondria known as kinetoplast), each species has its own insect vector, particular life-cycle features, different target tissues, and distinct disease pathogenesis in mammalian hosts (Jackson 2015).

The genomes of *T. cruzi*, *T. brucei*, and *L. major* have been initially sequenced and compared with better understand gene evolution and genetic variation in these related pathogens (Ghedin et al. 2004; El-Sayed, Myler, Blandin, et al. 2005). A remarkable finding derived from the comparative analysis of Tritryps genomes was the great number of shared genes (El-Sayed, Myler, Blandin, et al. 2005). However, the repetitive DNA was extremely different in these species. Repetitive DNA sequences are scarce in the *L. major* genome, but comprises up to half of the *T. cruzi* genome. Moreover, *L. major* is believed to be devoid of active transposable elements (TEs) (Ghedin et al. 2004; Ivens et al. 2005; Bringaud et al. 2006), but both *T. cruzi* and *T. brucei* genomes harbor intact and autonomous TEs (Wickstead et al. 2003; El-Sayed, Myler, Bartholomeu, et al. 2005; Bringaud et al. 2008; Thomas et al. 2010; Berná et al. 2018). Caused by this intrinsic genome complexity—abundance of repetitive sequences and genes organized in tandem—the *T. cruzi* genome remained fragmented even through long-read sequencing (1,142 and 599 scaffolds in hybrid and nonhybrid strains, respectively; Berná et al. 2018), and all of the *T. cruzi* sequencing projects based on short reads have demonstrated that genome assembly and downstream comparative analyses are extremely challenging in this species.

Genome annotation procedures are mainly focused on standard genetic elements, frequently neglecting repetitive sequences due to their hard-achieving de novo assembly (Treangen and Salzberg 2012). As a consequence, repetitive DNA is poorly described and studied (Altemose et al. 2014). In this context, RepeatExplorer has emerged as a widely used approach to comprehensively evaluate the nature of repetitive sequences. This bioinformatic tool attempts to cluster low-coverage high-throughput sequencing reads using a graph-based algorithm to characterize and quantify the complete repetitive DNA fraction of a genome (Novák et al. 2010, 2013, 2017), which nowadays is known as the “repeatome” (Maumus and Quesneville 2014). Low-coverage sequencing is a cost-effective approach that does not require having previous information about the target genome and avoids dealing with whole-genome assemblies. Beyond RepeatExplorer was originally conceived to analyze plant repeatomes, it has been successfully applied in mammals (Pagán et al. 2012), insects (Ruiz-Ruano et al. 2016; Palacios-Gimenez et al. 2017; Pita et al. 2017), and fishes (Utsunomia et al. 2017). Here, we used low-coverage sequencing and the RepeatExplorer approach to compare the repeatomes of *T. cruzi*, *T. brucei*, and *L. major* to reference genomes. Four genomes of *T. cruzi*—with different sequencing technology approaches—were compared. CL Brenner strain with BAC-end Sanger sequencing (El-Sayed, Myler, Bartholomeu, et al. 2005), Sylvio X10 strain with 454 technology (Franzén et al. 2011), and the newly less collapsed PacBio sequenced strains Dm28c and TCC (Berná et al. 2018). The extensive amplification of repeated DNA is the main reason why the *T. cruzi* genomes were very poorly assembled. Since firsts genomes of *T. brucei* and *L. major* are considered as high quality assemblies, those were used as reference (Berriman et al. 2005; Ivens et al. 2005).

**Fig. 1.**
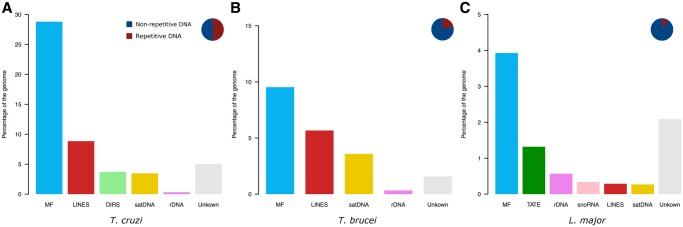
—Comparison of *Trypanosoma cruzi*, *T. brucei*, and *Leishmania major* repeatomes. Bar plots show the relative amount of each repetitive DNA fraction on the (*A*) *T. cruzi*, (*B*) *T. brucei*, and (*C*) *L. major* genome. Pie charts represent the relative amount of repetitive and nonrepetitive DNA on each genome.

**Fig. 2.**
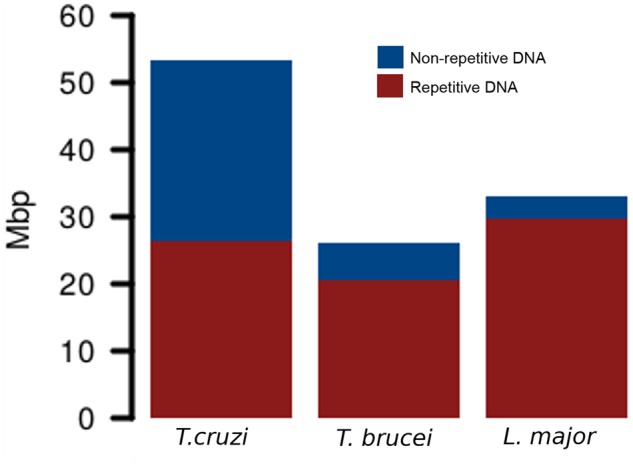
—Comparison of *Trypanosoma cruzi*, *T. brucei*, and *Leishmania major* genomes. Bar plots represent the total genome content in Mega base pairs (Mbp). In each genome, repetitive and nonrepetitive DNA is depicted.

First, kinetoplast DNA (mini- and maxicircles) was removed from raw Illumina reads and after quality filtering a random subsampling was performed to obtain ∼1× coverage in each genome. This resulted in 353,334 reads from *T. cruzi*, 173,836 reads from *T. brucei*, and 218,778 reads from *L. major* that were subsequently used in the RepeatExplorer analyses. The software initially identified 293, 203, and 199 clusters for *T. cruzi*, *T. brucei*, and *L.major*, respectively. In *T. cruzi*, we estimated that 51.25% of the genome corresponds to repetitive DNA sequences. Out of them, 28.81% were annotated as coding sequences belonging to multigenic families, 8.85% as LINEs (Long Interspersed Elements), 3.73% as DIRS-like or tyrosin recombinase (YR) elements (mostly VIPER element), 3.48% as satellite DNA, 0.31% represented ribosomal DNA (rDNA), and 5.07% remained as undetermined repeats. Conversely, in *T. brucei*, only 20.69% of the genome harbors repetitive DNA sequences. Out of them, we were able to determine that 9.53% belong to coding sequences from multigenic families, 5.67% to LINE TEs, 3.59% were satellite DNA repeats, 0.33% as rDNA, and 1.57% of the genome remained as undetermined repeats. Finally, the repetitive DNA fraction in *L. major* was smaller than in the genus *Trypanosoma*, corresponding only to 8.80% of the genome. The vast majority of this repetitive DNA consisted in multigenic families (see details later), which reached the 3.93% of the genome. Additionally, 1.32% was identified as TEs named telomere-associated mobile elements (TATEs), 0.29% as LINE TEs, and 0.27% assigned to satellite DNA repeats. In addition, several clusters belonged to rDNA genes and snoRNA regions, which accounted for the 0.57% and 0.34% of the genome, respectively. The remaining 2.09% of the genome was annotated as undetermined repeats (fig. 1). In terms of quantitative comparison, figure 2 is representing the total amount of genome content in mega base pairs (Mbp), depicting the repetitive and nonrepetitive sequences. Although difference in genome size on Tritryps is highly influenced by the repetitive DNA content, it does not the only responsible, since nonrepetitive DNA fraction abundance is quite different in each genome.

In agreement to previous quantification of repetitive DNA in the genomes of *T. cruzi* CL Brener (El-Sayed, Myler, Bartholomeu, et al. 2005), Sylvio X-10 (Franzén et al. 2011), Dm28c and TCC (Berná et al. 2018) strains, our current analysis showed that almost half of the genome is composed by these sequences. Within the repetitive fraction, the most abundant sequences correspond to multigenic families as previously described on reference genomes. However, the relative abundance of each family remained uncertain and probably underestimated (El-Sayed, Myler, Bartholomeu, et al. 2005). It was only with the upcoming of the new referenced genomes (Berná et al. 2018), that we were able to compare our measure data with a noncollapsed genome, rendering quite similar quantification of the multigenic families as a whole. Still, it seems that our methodology deals with pseudogenes better that the classical methods for annotating multigenic families in an assembled genome. Since RepeatExplorer clusterization merge them together, more pseudogenes are annotated. On the other hand, we are not able to separate between proper genes and pseudogenes, but this can not be the objective using Illumina reads. Here, we were able to quantify the relative abundance of trans-sialidase (TS), retrotransposon hot spot (RHS), mucins, mucin-associated surface proteins (MASP), surface protein dispersed gene family-1 (DGF-1) and GP63 multigenic families (fig. 1). The amount of these families vary among different *T. cruzi* strains, as has been shown between CLBrener and X-10 Sylvio, which could be related to their infection capacity, since these protein-coding genes are involved in the parasite–host interactions (Franzén et al. 2011). The application of low-coverage sequencing and RepeatExplorer analysis over multiple *T. cruzi* strains with differential infectivity may uncover the relationship between multigenic dynamics and pathogenesis. TEs ranked second in terms of abundance being almost 13% of the genome. LINEs (such as the L1Tc, NARTc, CZAR, and TcTREZO elements) where more abundant than YR elements (VIPER element and their nonautonomous derivative SIRE). These repetitive DNA sequences were also underestimated on previous analyses (El-Sayed, Myler, Bartholomeu, et al. 2005; Franzén et al. 2011; Berná et al. 2018). As explained for multigenic families, TE fragments are not usually identified when classical procedures are done. Nevertheless, it must been taken into account that TEs richness differences between strains has been already described (Vargas et al. 2004). It could be attributed to natural variations between *T. cruzi* strains, but considering the remarkable disparity (5% estimated for CL Brener) this must deserve further attention, since additional factors than strain diversification may be explaining TEs dynamics. Moreover, TEs quantification on both newly generated reference genomes showed almost the same TEs abundance for TCC strain—closely related to CL Brenner—and Dm28c strain (Berná et al. 2018), evidencing that RepeatExplorer is a valid tool for TEs recognition and quantification. Lastly, satellite DNA sequences encompass >3% of the genome, being vastly dominated by the 195-nt satellite. Previous 195-nt satellite quantification on CL Brenner estimated that 5% of the genome is composed by this repeat (Martins et al. 2008). However, variation of 195-nt abundance has been reported to be 4- to 6-fold between DTU TcI and DTU TcII strains (Elias et al. 2003; Vargas et al. 2004). This difference is also observed in the new reference genomes from TCC and Dm28c. Actually, the Dm28c quantification is close to that reported here, reinforcing that low-coverage sequencing provides reproducible estimations of repeat element abundances. Several other tandem repeats have been recently described (Berná et al. 2018) but only a few of them were retrieved by RepeatExplorer, indicating that their abundances are below the threshold set for a standard analysis. However, we aimed to render a coarse-grain, genome-wide overview rather than a meticulous description of all repeats.

*Trypanosoma brucei* genome is composed by ∼20% of repetitive DNA. Similar to *T. cruzi*, multigenic families were the most abundant repeats reaching ∼10% of the genome, with RHS and VGS/ESAG as the most representative families. TEs in *T. brucei* represented 5.67% of the genome, but in this case is only composed by LINE sequences, such as the *Tbingi* elements, its related nonautonomous RIMEs, and a few SLAC elements. Although VIPER elements are described in *T. brucei* (Lorenzi et al. 2006), these repeats are known to be in very low copy number, hence undetectable under our approach. The first draft genome of *T. brucei* presented in 2005 (Berriman et al. 2005) only reported that subtelomeric genes were just over 20% and that TEs represented 2% of the genome (El-Sayed, Myler, Bartholomeu, et al. 2005), however, nothing is said about the satellite DNA. Our results showed two prominent satellite DNA families, the 177-bp repeat described to be part of intermediate and minicromosomes which are enriched by VSG genes (Sloof et al. 1983; Wickstead et al. 2004; Obado et al. 2005), and the 147-bp repeat (named CIR147) present in the centromere form the majority of macrochromosomes (Obado et al. 2007).

**Fig. 3.**
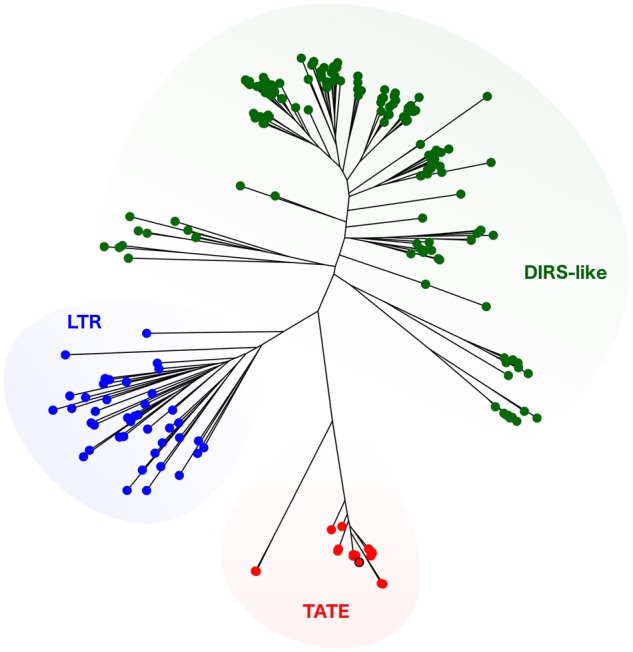
—Phylogenetic characterization of TATE elements. Maximum-likelihood phylogenetic tree using full retrotranscriptase domain sequences from all Trypanosomatidae TATE reported hitherto, and several DIRS-like elements retrieved from databases. Other LTR elements were used as outgroup. The *Leishmania major* TATE consensus sequence is marked by a black edge.

The most surprising results came along with the TEs analysis in *L. major*. Repetitive DNA comprehends <10% of the genome, as was expected since former genome analyses described smaller subtelomeric regions than in *Trypanosoma* species (Ivens et al. 2005; Peacock et al. 2007). Furthermore, the closely related *L. braziliensis* and *L. infantum* have also ∼10% of the genome composed by DNA repeats (Peacock et al. 2007). Although the reference genome for *L. infantum* has been resequenced using long-reads technology, revealing an expansion of coding genes copy number, the amount of repetitive DNA was not cited (González-De La Fuente et al. 2017). As observed in *Trypanosoma*, the majority of the repeated genome was represented by gene-coding sequences, being GP-63 and the Leucin-rich repeats among the most abundant elements. Remarkably, as in *L. braziliensis* genome (Peacock et al. 2007) but not described so far for *L. major*, we found traces of a the LINE element related to CRE2 (from *Crithidia fasciculata*), which is also related with CZAR and SLACs TEs from *T. cruzi* and *T. brucei*, respectively. Another interesting finding was the presence of a truncated element bearing a reverse transcriptase domain, from the LINE order. This probably corresponds to the LmDIRE elements, which are included in the *ingi2* clade (Bringaud et al. 2009). By far, an exceptional finding was the recovering of TATE copies representing 1.32% of the genome. These elements were previously reported in other *Leishmania* species from the subgenus *Viannia*, such as *L. braziliensis* (Peacock et al. 2007) and *L. panamensis* (Llanes et al. 2015), but not from the subgenus *Leishmania*, as *L. major*. Sequence similarity searches on the *L. major* genome available on TriTrypdb (https://www.TriTrypdb.org) did not retrieve any positive results. Currently, TATEs are not classified within any of the TEs families, nor even as a concrete class. However, the presence of a tyrosine recombinase suggests that possibly TATEs are DIRS-like TEs (Peacock et al. 2007). Here, we were able to reconstruct a consensus sequence for the retrotranscriptase domain of the *L. major* TATE element, and determine that all kinetoplastid TATEs described hitherto form a separated clade from other DIRS-like elements (fig. 3). Further analysis on these elements would be of major interest for better understanding the dynamics of *Leishmania* genomes. Beyond the already know impact of TEs in trypanosomatid genomes (Bringaud et al. 2008; Thomas et al. 2010), our finding that TATE elements account for a considerable part of the *L. major* genome, could change the evolutionary paradigm of a genome that was believed to be almost TE-free. Actually, it has been already suggested that TATEs are not restricted to telomeric regions in *L. panamensis* genome, and that they could be playing a central role in gene regulation and structuring (Llanes et al. 2015). For example, being candidates to participate on recombinational events leading to genetic amplification (Ubeda et al. 2014). Genome localization of TATE elements within *L. major* genome could not be determined by our methodology; new assemblies from long reads would be the best approach on this issue.

In conclusion, we have shown that our results are comparable to those obtained for other Tritryps strains and implementing different sequencing strategies, such as high-coverage and long-read genomic assemblies. This supports that our method using low-coverage, Illumina short reads is useful for a genome-wide characterization of trypanosomatid repeatomes, and could be useful to perform comparative analyses of the repetitive DNA repertories in other protozoan species. Noteworthy, our strategy allowed to identify genetic features that were not described so far, such as TATEs elements in the *L. major* genome.

## Materials and Methods

### Strains and DNA Purification

*Trypanosoma* *cruzi* Dm28c (Contreras et al. 1988) epimastigotes were cultured axenically in liver infusion tryptose medium supplemented with 10% (v/v) inactivated fetal bovine serum (GIBCO, Gaithersburg, MD) at 28 °C. *Leishmania major* (FRIEDLIN strain) and *T. brucei* (TREU927 strain) were cultured in modified RPMI medium containing 10% (v/v) inactivated fetal bovine serum (GIBCO, Gaithersburg, MD) at 28 °C. Quick-DNA Universal kit (Zymo Research, Irvine, CA) was used according to the manufacturer’s specifications for isolation of genomic DNA in logarithmic growth phase. The DNA was resuspended in sterile distilled water and stored at 4 °C until use. Quantification was performed using Qubit™ dsDNA HS Assay Kit (Invitrogen by Thermo Fisher Scientific, San Jose, CA).

### Illumina Sequencing and Bioinformatic Analyses

Genomic libraries were prepared with the Nextera XT DNA Sample Preparation Kit (Illumina, San Diego, CA), analyzed using 2100 Bioanalyzer (Agilent Technologies, Palo Alto, CA), and then sequenced using a MiSeq Illumina platform, which produced 540831, 1790895, and 1322286 pair-end reads (2 ×150 cycles) for *T. cruzi*, *T. brucei*, and *L.major*, respectively. Low-coverage sequencing data results are available on SRP155233.

Kinetoplast DNA (mini- and maxicircles) was removed from raw Illumina reads, using DeconSeq (Schmieder and Edwards 2011) with a custom database made from several kinetoplastid maxicircles sequences deposited in GenBank. Quality filtering was performed with TRIMMOMATIC (Bolger et al. 2014) under LEADING: 3 TRAILING: 3 SLIDINGWINDOW: 4 : 20 MINLEN: 149 parameters. The random subsampling was performed to obtain ∼1× coverage in each genome with the shuf bash command. Graph-based clustering analyses were carried on separately using RepeatExplorer default options, implemented within the Galaxy environment (http://repeatexplorer.org/) (Novák et al. 2010, 2013, 2017). Cluster annotation was supported with a custom database of repeated gene families, retroelements, and satDNA, based on the newly PacBio sequenced reference genome annotation (Berná et al. 2018).

*Leishmania* *major* TATE consensus sequence was determined assembling the raw reads which belonged to RepeatExplorer clusters annotated as TATEs. Assembly of these reads was performed using CAP3 (Huang and Madan 1999), following a hand curation of the sequence alignment using SeaView (Gouy et al. 2010). TATE elements from other kinetoplastid genomes (*Bodo saltans, Leptomonas pyrrhocoris, T. theileri, L. braziliensis, Angomonas deanei*) were retrieved from NCBI, using BLAST search using the *L. major* TATE consensus sequence as query. DIRS-like sequences were downloaded from Repbase (https://www.girinst.org/repbase/) and only those with complete retrotranscriptase domains were used. DIRS-1 retrotranscriptase domain sequences were also recovered from the GenBank cd03714 sequence cluster, and retrotranscriptase domain sequences from other LTR elements were used as outgroup (cd01647 sequence cluster). Alignment of amino acid sequences was performed using MAFFT software (Katoh et al. 2002) under the G-INS-i method. Phylogenetic reconstruction was performed with PhyML (Guindon et al. 2010) under the WAG substitution model and the aLRT (Shimodaria–Hasegawa-like) test was employed for internal node support. 

## Footnotes

**Data deposition:** This project has been deposited at NCBI under the accesion SRP155233
